# Parental Smoking and Risk of Childhood-onset Type 1 Diabetes

**DOI:** 10.1097/EDE.0000000000000911

**Published:** 2018-09-28

**Authors:** Maria C. Magnus, German Tapia, Sjurdur F. Olsen, Charlotta Granstrom, Karl Mårild, Per M. Ueland, Øivind Midttun, Jannet Svensson, Jesper Johannesen, Torild Skrivarhaug, Geir Joner, Pål R. Njølstad, Ketil Størdal, Lars C. Stene

**Affiliations:** From the a Centre for Fertility and Health, Norwegian Institute of Public Health, Oslo, Norway; b MRC Integrative Epidemiology Unit at University of Bristol, Bristol, United Kingdom; c Department of Population Health Sciences, Bristol Medical School, Bristol, United Kingdom; d Division of Mental and Physical Health, Norwegian Institute of Public Health, Oslo, Norway; e Centre for Fetal Programming, Department of Epidemiology Research, Statens Serum Institut, Copenhagen, Denmark; f Department of Clinical Science, University of Bergen, Bergen, Norway; g Laboratory of Clinical Biochemistry, Haukeland University Hospital, Bergen, Norway; h Bevital AS, Bergen, Norway; i Department of Pediatrics, Copenhagen University Hospital, Herlev, Denmark; j Division of Pediatric and Adolescent Medicine, Oslo University Hospital, Oslo, Norway; k Institute of Clinical Medicine, University of Oslo, Oslo, Norway; l Department of Pediatrics and Adolescent Medicine, Haukeland University Hospital, Bergen, Norway; m KG Jebsen Center for Diabetes Research, Department of Clinical Science, University of Bergen, Bergen, Norway; n Department of Pediatrics, Østfold Hospital Trust, Grålum, Norway.

**Keywords:** childhood type 1 diabetes, father, mother, nicotine, smoking

## Abstract

**Background:**

A few prospective studies suggest an association between maternal smoking during pregnancy and lower risk of type 1 diabetes. However, the role of unmeasured confounding and misclassification remains unclear.

**Methods:**

We comprehensively evaluated whether maternal smoking in pregnancy predicts lower risk of childhood-onset type 1 diabetes in two Scandinavian pregnancy cohorts (185,076 children; 689 cases) and a Norwegian register-based cohort (434,627 children; 692 cases). We measured cord blood cotinine as an objective marker of nicotine exposure during late pregnancy in 154 cases and 476 controls. We also examined paternal smoking during pregnancy, in addition to environmental tobacco smoke exposure the first 6 months of life, to clarify the role of characteristics of smokers in general.

**Results:**

In the pregnancy cohorts, maternal smoking beyond gestational week 12 was inversely associated with type 1 diabetes, pooled adjusted hazard ratio (aHR) 0.66 (95% CI = 0.51, 0.85). Similarly, in the Norwegian register-based cohort, children of mothers who still smoked at the end of pregnancy had lower risk of type 1 diabetes, aHR 0.65 (95% CI = 0.47, 0.89). Cord blood cotinine ≥30 nmol/L was also associated with reduced risk of type 1 diabetes, adjusted odds ratio 0.42 (95% CI = 0.17, 1.0). We observed no associations of paternal smoking during pregnancy, or environmental tobacco smoke exposure, with childhood-onset type 1 diabetes.

**Conclusion:**

Maternal sustained smoking during pregnancy is associated with lower risk of type 1 diabetes in children. This sheds new light on the potential intrauterine environmental origins of the disease.

## Abstract 2

Supplemental Digital Content is available in the text.

Together with known genetic influences, unidentified early environmental factors likely play a role in development of childhood-onset type 1 diabetes.^1,2^ Previous studies report inverse,^3–7^ positive,^8^ and null^9,10^ associations between maternal smoking during pregnancy and type 1 diabetes in the offspring. Interestingly, most prospective studies suggest a lower risk of type 1 diabetes in offspring of mothers who smoked during pregnancy.^2^ One small study also indicated an inverse association between paternal smoking and type 1 diabetes.^10^

The authors of previous studies indicating an inverse association between maternal smoking during pregnancy and risk of childhood-onset type 1 diabetes suggested unmeasured confounding and misclassification owing to reliance on self-reported information, as potential explanations.^3–5^ These studies all lacked an objective measure of exposure to maternal smoking. The current weak evidence for similar inverse associations of both maternal and paternal smoking when the mother was pregnant with childhood-onset type 1 diabetes suggests that the associations might reflect unmeasured confounding by background characteristics linked to smoking in both parents.^11,12^

To test the hypothesis that maternal smoking during pregnancy is associated with a lower risk of type 1 diabetes, we wanted to further examine the role of unmeasured confounding and misclassification, using two of the world’s largest pregnancy cohorts and a national register-based cohort. To examine the role of unmeasured confounding, we estimated the associations of paternal smoking and maternal smoking only before pregnancy with type 1 diabetes as negative controls.^13^ To examine the potential role of recall bias attributable to self-report of maternal smoking during pregnancy, we also measured cord blood cotinine as a biomarker of exposure to nicotine during late pregnancy in a subsample of one of the pregnancy cohorts.

## METHODS

### The Norwegian Mother and Child Cohort Study and the Danish National Birth Cohort

The Norwegian Mother and Child Cohort Study (MoBa) recruited pregnant women across Norway between 1999 and 2008, at approximately gestational week 18.^14,15^ Of the eligible women, 41% participated, and all participants gave written informed consent. The cohort includes more than 95,000 women and 114,500 children. We used data available in March 2014 (version VIII). A total of 98,287 singletons in MoBa with information from questionnaire administered at gestational week 18 were included in the current study (eFigure 1 a; http://links.lww.com/EDE/B394). The data collection in MoBa is approved by the Norwegian Data Inspectorate, and the current subproject is approved by the Regional Ethics Committee for Medical Research of South East Norway.

The Danish National Birth Cohort (DNBC) recruited pregnant women at their first antenatal visit across Denmark between 1996 and 2002.^16,17^ Approximately 50% of all general practitioners in Denmark participated in the recruitment, and 60% of invited women agreed to participate, resulting in 91,745 mothers and 103,118 children. All participants provided written informed consent. A total of 86,789 singletons from DNBC with information gathered through telephone interview at gestational week 12 were included in the current study (eFigure 1 b; http://links.lww.com/EDE/B394). The data collection in the DNBC is approved by the Danish National Ethics Board.

### Norwegian National Registry Cohort

We also analyzed maternal smoking during pregnancy and childhood-onset type 1 diabetes in a Norwegian national register-based cohort. The linkage included information from the Medical Birth Registry of Norway, the Norwegian Prescription Database, and the Norwegian Patient Registry, in addition to information on maternal education from Statistics Norway. The registry linkage included information on all deliveries in Norway between January 2004, when the prescription registry was set up, and December 2012 (n = 541,036). After exclusion of children with a birth weight of 500 g or less (n = 419), gestational age of 22 weeks or less (n = 3,818), children with missing information on maternal smoking during pregnancy (n = 99,546), and children with missing maternal identification number (n = 2,567), 434,686 children were eligible for the analysis. The national register linkage is approved by the Norwegian Data Inspectorate and the Regional Ethics Committee for Medical Research of South East Norway.

### Parental and Household Smoking Based on Self-report

For the two pregnancy cohorts, we obtained self-reported information on parental frequency and amount of smoking before pregnancy (MoBa only), during pregnancy, and the first 6 months of the child’s life, in addition to whether the child spent time in a room where someone was smoking the first 6 months of life. Maternal smoking during pregnancy was categorized as none (reference), only during the first 12 gestational weeks, and also after gestational week 12. In a secondary analysis in MoBa, we distinguished the reference category according to mothers who had never smoked and mothers who only smoked before pregnancy. We also did an exploratory analysis of the average number of cigarettes smoked per day during pregnancy, categorized as none, unknown (but reported smoking during pregnancy), ≤5, 6–10, and more than 10. Additional information on exposure to paternal ([or] maternal partner) smoking the first 6 months of life was available for MoBa. In the analysis of the Norwegian register-based cohort, information on maternal smoking during pregnancy was obtained through the birth registry and was categorized as none, only at the beginning of pregnancy, and still smoking at the end of pregnancy based on self-report at the time of delivery.

### Cord Blood Cotinine Levels

We measured cord blood cotinine levels in 154 cases with type 1 diabetes and 476 randomly selected controls in MoBa. The sampling, processing, and storage conditions in the MoBa biobank have been described previously.^18^ The BEVITAL laboratory, Bergen, measured cord blood cotinine levels in plasma using liquid chromatography–tandem mass spectrometry.^19^ Cotinine levels were categorized as less than 1 nmol/L (not detectable), 1–29 nmol/L, and ≥30 nmol/L. A cotinine level ≥30 nmol/L has previously shown a high correspondence with self-reported daily smoking among MoBa mothers.^20^

### Childhood-onset Type 1 Diabetes

The outcome in the two pregnancy cohorts (and nested case–control study) was a clinical diagnosis of type 1 diabetes, ascertained by national Childhood Diabetes Registers, which are nationwide registers with a high level of case ascertainment.^21,22^ Information on new cases of type 1 diabetes are reported from hospitals to the registers, and the date of first insulin treatment is used to define the age of onset. Rare cases of type 2 diabetes or monogenic diabetes were excluded from both registers. For the analysis in the Norwegian register-based cohort, childhood-onset type 1 diabetes was defined based on dispensed prescriptions for insulin in the prescription registry (Anatomical Therapeutic Chemical Classification System code A10A) and/or a diagnosis of type 1 diabetes in the patient registry (International Classification of Diseases version 10 code E10). Individuals with only one registration of the International Classification of Diseases version 10 code E10, without any dispensed insulin in prescription registry, were excluded from analyses (n = 59) owing to their uncertain case status.

### Covariates

Information on parental age, maternal parity, child sex, and birth weight were obtained from national birth registers. In addition, parental education and body mass index (weight kg/height m^2^) were obtained through questionnaires/interviews in the two pregnancy cohorts. Finally, information on parental history of type 1 diabetes was obtained through the patient registry for MoBa participants, whereas maternal history of all types of diabetes was gathered through a national diabetes registry for DNBC participants.^23^ For the Norwegian register-based cohort, maternal insulin-treated diabetes was defined as two or more dispensed prescriptions for insulin in the prescription registry, and maternal educational level was available from statistics Norway. Participants in the case–control study of cord blood cotinine levels were also genotyped using a custom Illumina Golden Gate assay (Illumina, San Diego, CA). DNA extraction, genotyping methods, and quality control procedures have been described previously (see Ref. ^24^; details in supplementary Data; http://links.lww.com/EDE/B394). tag-SNPs (n = 144) on chromosome 6 were used to impute human leukocyte antigen (HLA) class II genotype^25^ and confirmed by classical HLA genotyping using allele-specific polymerase chain reaction. HLA genotypes were categorized into two groups based on established risk for type 1 diabetes: increased risk (at least one copy of HLA DQA1*03-DQB1*03:02 [DQ8-DR4] or DQA1*05:01-DQB1*02:01 [DQ2-DR3], and no protective HLA-DQA1*01:02-DQB1*06:02 haplotype) or no increased risk (any other genotype).

### Statistical Analysis

We estimated the associations of parental and household smoking with childhood-onset type 1 diabetes using Cox proportional hazards regression, reporting hazard ratios (HR) and 95% confidence intervals (CI). We combined the results from the two pregnancy cohorts in a random-effects meta-analysis. Heterogeneity between the cohort-specific estimates was examined by the *I*^2^ statistic. For the analysis of parental smoking during pregnancy, children were followed from date of birth until type 1 diabetes diagnosis or the end of follow-up (21 July 2016 for MoBa, 15 May 2016 for DNBC, and 31 December 2014 for the Norwegian registry-based cohort). In the analysis of environmental tobacco smoke exposure during the first 6 months of life, the participants were followed from when they were 6 months of age. We used a robust cluster variance estimator to account for the presence of siblings. The proportional hazards assumption was examined using the Schoenfeld residuals and testing the interaction with time. We used logistic regression to estimate the association between cord blood cotinine levels and childhood-onset type 1 diabetes in the MoBa case–control sample, reporting odds ratios (OR) and 95% CI.

The multivariable analysis of maternal smoking during pregnancy and childhood-onset type 1 diabetes were adjusted for the mother’s age, parity, education, prepregnancy body mass index, and diabetes, whereas in analyses of paternal smoking during pregnancy, we adjusted for the father’s age, education, body mass index, and type 1 diabetes. These covariates were all considered to be confounders, although we acknowledge that smoking and body mass index have a complicated bidirectional relationship. For the analysis of cord blood cotinine levels and risk of type 1 diabetes, we were also able to adjust for HLA genotype as a potential confounder. With regard to parental smoking during the first 6 months of the child’s life, we further adjusted for parental smoking during pregnancy, in addition to the background characteristics described above. The analysis of the Norwegian register-based cohort adjusted for maternal age, parity, education, and type 1 diabetes. We also examined the role of mediation by delivery by cesarean section, birthweight, and weight gain the first year of life for the association between maternal smoking during pregnancy and type 1 diabetes, by including these covariates in the multivariable model. This mediator model also adjusted for offspring sex as a potential confounder of the association between the pregnancy complications and type 1 diabetes.

The amount of missing information for the individual covariates ranged between 0% and 17%. We imputed in total 20 datasets containing missing covariate information, using multiple imputation by chained equations, including all of the variables in the analysis model into the imputation model, in addition to the baseline hazard. We conducted the analysis using SAS version 9.4 (SAS Institute, Cary, NC) and Stata version 14 (StataCorp, College Station, TX).

## RESULTS

A total of 185,076 children born between February 1998 and July 2009 from MoBa and DNBC were available for the primary analysis after multiple imputation of missing exposure and covariate information (eFigure 1; http://links.lww.com/EDE/B394). The mean age of the children at the end of follow-up was 11.0 years (range: 7.0–17.5 years) in MoBa and 15.5 years (range: 13.0–18.2 years) in DNBC. The incidence rate of type 1 diabetes was 33 per 100,000 person-years in MoBa and 28 per 100,000 person-years in DNBC. Overall, the background characteristics in the two cohorts were similar (Table 1). The distribution of characteristics was similar among children included in the analysis after multiple imputation of missing covariate information and among children included in the complete case analysis (eTable 1; http://links.lww.com/EDE/B394).

**TABLE 1.**
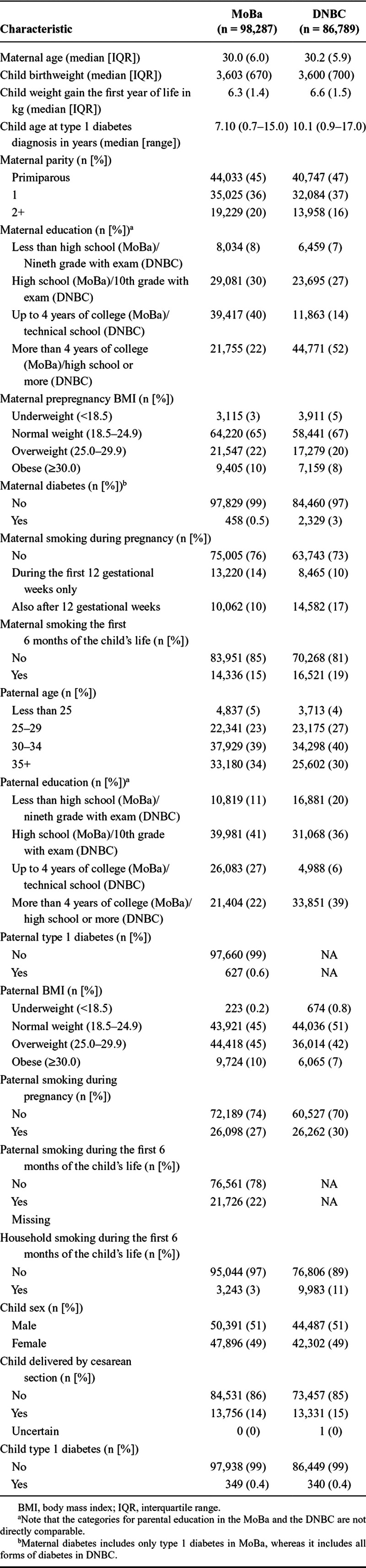
Distribution of Background Characteristics in the MoBa and the DNBC

### Parental Smoking During Pregnancy and Childhood-onset Type 1 Diabetes in Two National Pregnancy Cohorts

eFigure 2 (http://links.lww.com/EDE/B394) shows the risk of type 1 diabetes by maternal smoking during pregnancy in MoBa and DNBC. For both cohorts combined, maternal sustained smoking during pregnancy was associated with a reduced risk of childhood-onset type 1 diabetes, with a pooled adjusted HR of 0.66 (95% CI = 0.51, 0.85) when comparing children of mothers who still smoked after gestational week 12 to children of mothers who did not smoke (Table 2). In contrast, paternal smoking during pregnancy was not associated with childhood-onset type 1 diabetes, with a pooled adjusted HR of 0.97 (95% CI = 0.82, 1.2; Table 2). The inverse association between maternal smoking and childhood-onset type 1 diabetes was not mediated by birth weight, delivery by cesarean section, nor by infant weight gain the first year of life (eTable 2; http://links.lww.com/EDE/B394). The complete case analysis yielded similar associations (eTable 3; http://links.lww.com/EDE/B394). When we evaluated the association between the average number of cigarettes the mother smoked per day during pregnancy and childhood-onset type 1 diabetes, the estimates indicated some evidence for a dose–response relationship, but the modest number of cases within each exposure group yielded imprecise confidence intervals (eTable 4; http://links.lww.com/EDE/B394). Interestingly, maternal smoking only before pregnancy, that is, no intrauterine exposure, showed no association with type 1 diabetes in MoBa, adjusted HR 0.95 (95% CI = 0.73, 1.2). We observed some deviation from the proportional hazard assumption for maternal smoking during pregnancy in DNBC (*p* value for interaction with time 0.02), but the same was not observed for MoBa (*p* value >0.1).

**TABLE 2.**
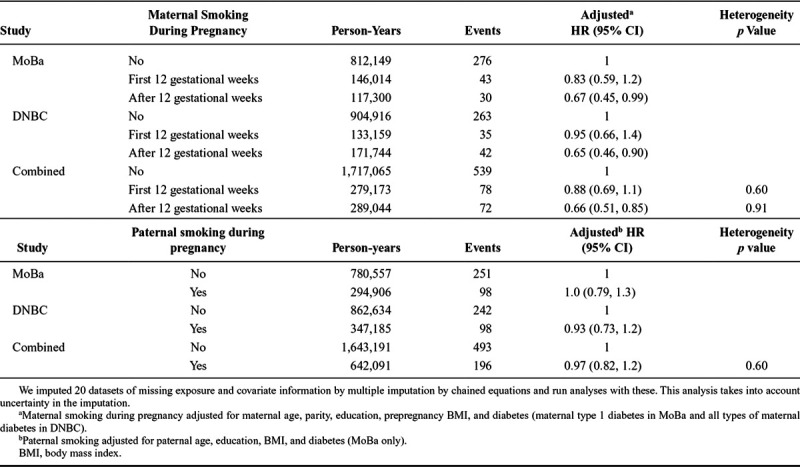
Associations of Parental Smoking During Pregnancy and Childhood-onset Type 1 Diabetes in the MoBa and the DNBC

### Environmental Tobacco Smoke Exposure the First 6 Months of the Child’s Life and Childhood-onset Type 1 Diabetes

There was no strong evidence that exposure to maternal smoking during the first 6 months of life, pooled adjusted HR 0.78 (95% CI = 0.55, 1.1), nor exposure to household smoking during the first 6 months of life, pooled adjusted HR 1.2 (95% CI = 0.83, 1.6), were associated with childhood-onset type 1 diabetes (Table 3). The results were similar in the complete case analysis (eTable 5; http://links.lww.com/EDE/B394). In line with these findings, exposure to paternal smoking during the first 6 months of life also showed no strong evidence of an association with childhood-onset type 1 diabetes in MoBa (eTable 6; http://links.lww.com/EDE/B394). There was also some evidence for a deviation from the proportional hazard assumption for the association between exposure to household smoking in MoBa (*p* value for interaction with time 0.03), but the same was not observed for DNBC (*p* value >0.1).

**TABLE 3.**
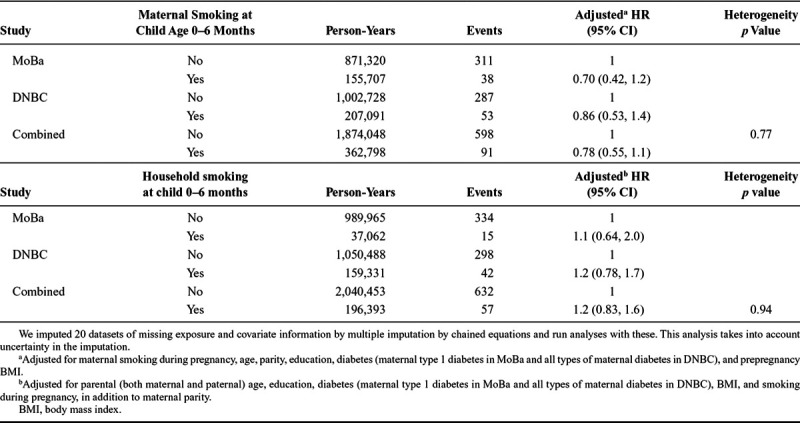
Associations of Environmental Tobacco Smoke Exposure During the First 6 Months of Life and Childhood-onset Type 1 Diabetes in the MoBa and the DNBC

### Maternal Smoking During Pregnancy and Childhood-onset Type 1 Diabetes in the Norwegian Register-based Cohort

The risk of childhood-onset type 1 diabetes was similar among those included (n = 434,627) in the analysis of the Norwegian register-based cohort, and those excluded owing to missing exposure information (n = 106,409), with a HR of 1.1 (95% CI = 0.96, 1.4). The mean age at the end of follow-up in the register cohort was 6.4 years (min 2.0, max 10.9). The overall incidence rate of type 1 diabetes among those included in the analysis was 25 per 100,000 person-years, and the mean age at onset was 4.7 years (min 0.2, max 10.5). The characteristics of the children included in the analysis are shown in eTable 7 (http://links.lww.com/EDE/B394). The risk of type 1 diabetes according to categories of maternal smoking is portrayed in eFigure 3 (http://links.lww.com/EDE/B394). Similar to the findings from the meta-analysis of the two pregnancy cohorts, children of mothers who still smoked at the end of pregnancy had decreased risk of type 1 diabetes, with an adjusted HR of 0.65 (95% CI = 0.47, 0.89; Table 4). There was no evidence that the association between maternal smoking during pregnancy and risk of type 1 diabetes was mediated by adverse pregnancy outcomes (eTable 8; http://links.lww.com/EDE/B394). Including the 59 children with uncertain case status in the analysis did not change our results.

**TABLE 4.**
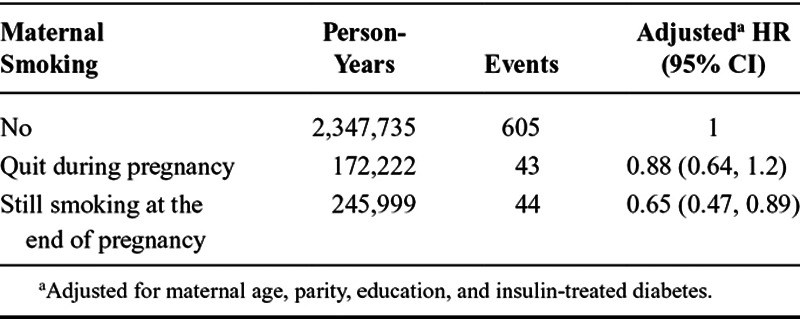
Association Between Maternal Smoking During Pregnancy and Childhood-onset Type 1 Diabetes in a Norwegian Register-based Cohort (n = 434,627)

### Cord Blood Cotinine Levels and Childhood-onset Type 1 Diabetes

The selection of the case–control sample with cord blood cotinine measures is illustrated in eFigure 4 (http://links.lww.com/EDE/B394). There was an inverse association between cotinine levels in cord blood and childhood-onset type 1 diabetes after adjustment for potential confounders, with an adjusted OR of 0.42 (95% CI = 0.17, 1.0) when comparing children with cotinine levels ≥30 nmol/L to those with nondetectable levels (Table 5). Additional adjustment for HLA genotype slightly attenuated the association. This result was similar (OR = 0.62; 95% CI = 0.22, 1.7) if we adjusted for HLA conferred risk in four categories (DQ8 and 2, HLA DQ8 or DQ2 but not DQ6, at least one DQ6 haplotype, or remaining genotypes; eTable 9; http://links.lww.com/EDE/B394). The complete case analysis also showed similar findings (eTable 10; http://links.lww.com/EDE/B394).

**TABLE 5:**
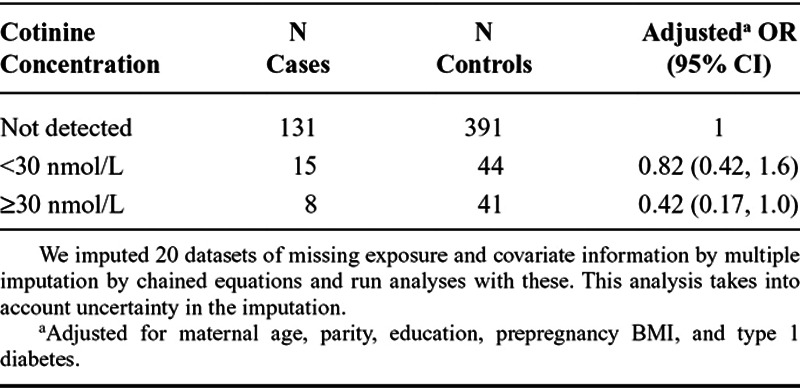
Associations of Cord Blood Cotinine Levels and Risk of Type 1 Diabetes in the Case–Control Study Nested in the Norwegian Mother and Child Cohort Study

## DISCUSSION

In this study, based on two of the world’s largest pregnancy cohorts and a national register-based cohort, continued maternal smoking after the first trimester was associated with a reduced risk of childhood-onset type 1 diabetes. Based on our findings of no association with paternal smoking, and no association with maternal smoking only before pregnancy, we believe that there is no strong evidence of confounding by unmeasured background characteristics linked to smoking in both parents. Nor was the inverse association between maternal smoking during pregnancy and type 1 diabetes likely to be explained by recall bias as a result of self-report, owing to the similar decreased risk of type 1 diabetes observed with high cord blood cotinine levels.

Important strengths of this study include the size, confirmation in two homogeneous pregnancy cohorts and a national registry linkage, adjustment for a broad range of potential confounding factors, inclusion of paternal smoking when the mother is pregnant as a negative control, and the objective marker of exposure to nicotine (cotinine) during late pregnancy. The low participation rate in the two cohorts, in addition to loss to follow-up, might have resulted in a selection bias.^15,16^ We used multiple imputation to account for missing information on individual covariates to include the largest possible number of participants in our analysis. Furthermore, the association between maternal smoking during pregnancy and type 1 diabetes was similar when evaluated in the Norwegian register-based cohort, which included all deliveries in Norway within a given period, and this lends support to the notion that selection bias should not have greatly influenced the results of the two pregnancy cohorts. We observed some deviation from the proportional hazards assumption. Unfortunately, we did not have a sufficiently large number of cases to start dividing the follow-up time into intervals. Notably, the magnitude of the associations observed using logistic regression was similar. Despite some differences between the three cohorts in the way we were able to define maternal diabetes, multivariable adjustment for maternal diabetes did not change the estimated associations. We also acknowledge that our mediation analysis where we obtained an estimate of the conditional direct effect by including the potential mediators in the multivariable model might be biased if there are unmeasured confounders of the association between the exposure and mediator or the association between the mediator and the outcome.^26^ Based on the fact that we observed no difference in the associations before and after multivariable adjustment for the hypothesized mediators, we do not think that there was strong evidence to support mediation. It also seems unlikely that any collider bias would have perfectly nullified any evidence of mediation.

In a previously published validation study comparing maternal cotinine levels at approximately 18 gestational weeks to self-reported smoking status at the same time, the sensitivity and specificity for self-reported daily smoking, using 30 nmol/L as the cutoff concentration, were 82% and 99%, respectively.^20^ Furthermore, when we compared self-report of sustained smoking during pregnancy and cord blood cotinine levels, the sensitivity and specificity were 92% and 95%, respectively. Based on the fact that we observed similar associations of both maternal self-reported smoking during pregnancy and cord blood cotinine levels with type 1 diabetes, we do not think that recall bias explained the observed inverse association.

Our findings are in line with previous studies reporting an inverse association after multivariable adjustment between maternal smoking during pregnancy and childhood-onset type 1 diabetes.^3–7^ Most previous studies had a modest number of cases of type 1 diabetes, ranging between 196 and 361, except the first study that reported this association (with 2,757 cases of type 1 diabetes).^7^ The estimated association between maternal smoking during pregnancy and childhood-onset type 1 diabetes ranged between 0.76 (95% CI = 0.54, 1.1)^3^ and 0.37 (95% CI = 0.22, 0.64).^4^ In addition, there is evidence from four case–control studies of children with a genetic predisposition to type 1 diabetes, which all show some evidence of an inverse association between maternal smoking during pregnancy and islet autoimmunity, but the confidence intervals included the null value.^27–30^ The only previous study that found evidence for an increased risk of type 1 diabetes among children of mothers who reported smoking in pregnancy consisted of 344 cases and matched controls based on age and HLA haplotype.^8^ This study reported an almost four-fold increased risk of type 1 diabetes (OR: 3.9; 95% CI = 1.2, 12) among children of mothers who smoked 10 or more cigarettes per day during pregnancy, compared with children of nonsmoking mothers.^8^

Based on the positive association between maternal smoking during pregnancy and fetal death, we cannot exclude a potential selection bias attributable to the fact that we had to restrict our analysis to live births. If offspring who are miscarried/stillborn among smoking mothers would have had a greater risk of type 1 diabetes if they had survived, this could have contributed the inverse association between maternal continued smoking during pregnancy and type 1 diabetes. As indicated, multivariable adjustment for HLA genotype did not change the association between cord blood cotinine levels and risk of type 1 diabetes. Furthermore, if such selective loss of high-risk fetuses was a strong explanation for our findings, we would have expected to see similar inverse associations of maternal smoking before and after gestational week 12 with risk of type 1 diabetes. We, therefore, do not think that there is robust evidence to support that selective fetal death strongly influenced the observed associations.

The fact that we did not observe an association between paternal smoking and childhood-onset type 1 diabetes, nor an association with maternal smoking only before pregnancy (or only after), indicates that the association with maternal smoking during pregnancy could be due to an in utero effect, as opposed to unmeasured background characteristics associated with parental smoking,^11,12^ This evidence is in contrast to what was proposed from the only previous study of paternal smoking and childhood-onset type 1 diabetes, which included 65 cases across two British cohorts.^10^ Furthermore, the fact that we observed a similar association between cord blood cotinine levels and childhood-onset type 1 diabetes in the MoBa cohort suggests that self-reporting of maternal smoking during pregnancy is not a major driver of the association.

Despite the obviously problematic public health message of our findings, they support the possibility that maternal smoking during pregnancy may have a biologic influence on the risk of childhood-onset type 1 diabetes. Of note, the association was seen only for sustained smoking and not for those who stopped smoking early in pregnancy. This is in contrast to teratogens operating very early during development but similar to what is seen for effects of maternal smoking on birth weight.^31^ This indicates that the pathways involved are susceptible to modification in late gestation during rapid growth. Nicotine is known to have immune-suppressive actions.^32^ The possibility that these influences on the immune system could play a role in development of type 1 diabetes is supported by animal experiments, indicating that nicotine exposure reduces the incidence of type 1 diabetes by changing the profile of pancreatic cytokine expression from Th1 to Th2.^33^ Maternal smoking during pregnancy is also associated with a lower risk of preeclampsia,^34^ and adult smokers have reduced risk of Parkinson disease^35^ and ulcerative colitis.^36^ It is, therefore, interesting to speculate whether there might be any common mechanisms that might explain these observations.

Another possible biologic mechanism for an in utero effect of maternal smoking is differential DNA methylation.^37,38^ The top finding from the meta-analysis of multiple cohorts looking at maternal smoking and cord blood DNA methylation was cg05575921 in the aryl hydrocarbon receptor repressor (*AHRR).*^37^ *AHRR* suppresses signaling via the aryl hydrocarbon receptor, which may be involved in the regulatory T-cell and T-helper 17 function.^39,40^ Furthermore, a pathway analysis indicated that maternal smoking during pregnancy influenced pathways specific to T-cell function.^41^ Although the main underlying driver of many of the T-cell, eosinophil, and neutrophil pathway scores was *GFI1*, additional genes identified included *IL22* and *IL2RA.*^41^ Of relevance to our findings, a lower expression of *IL2RA* can influence immunity and potentially the risk of type 1 diabetes.^42,43^

Although our study shows robust evidence for an inverse association between maternal smoking during pregnancy and childhood type 1 diabetes, maternal smoking is known to be positively associated with several adverse pregnancy outcomes (such as stillbirth,^44^ preterm birth,^45^ low birthweight,^46^ and congenital malformations^47^) and chronic diseases (such as childhood asthma^48^ and overweight/obesity^49^). Any potential beneficial effect of maternal smoking on type 1 diabetes does not outweigh these potential harmful effects.

In conclusion, maternal sustained smoking during pregnancy is associated with lower risk of type 1 diabetes in children. This sheds new light on the potential intrauterine environmental origins of the disease. Further studies investigating the potential mechanisms underlying this association may lead to understanding of novel pathways for type 1 diabetes and/or targets for prevention of the disease.

## ACKNOWLEDGMENTS

We are grateful to participants of the Norwegian Mother and Child Cohort Study, participants of the Danish National Birth Cohort, and the Norwegian Childhood and Adolescent Diabetes Study Group.

## Supplementary Material
