# Supplementary material for: Parental Smoking and Risk of Childhood-onset Type 1 Diabetes
Source: Epidemiology. 2018 Sep 28;29(6):848–56. doi: 10.1097/EDE.0000000000000911 (PMC6169737; doi:10.1097/EDE.0000000000000911)

eTable 1 Distribution of characteristics among children with information from all the necessary follow-up questionnaires and children included after imputation

Characteristic	MoBa		DNBC	
	Information from all follow-up questionnaires used (n=81,630)	Children included after multiple imputation (n=98,287)	Information from all follow-up questionnaires used (n= 50,701)	Children included after multiple imputation (n=86,789)
Maternal age (Median(IQR))	30 (6)	30 (6)	30.6 (4.2)	30.2 (5.9)
Maternal parity (n(%))				
Primiparous	37,413 (46)	44,033 (45)	23,061 (45)	40,747 (47)
1	28,731 (35)	35,025 (36)	19,004 (37)	32,084 (37)
2+	15,486 (19)	19,229 (20)	8,621 (17)	13,958 (16)
Missing	0 (0)		15	
Maternal education (n(%)) [a]				
Less than high school (MoBa)/9th grade with exam (DNBC)	5,579 (7)	8,034 (8)	3,672 (7)	6,459 (7)
High school (MoBa) /10th grade with exam (DNBC)	23,452 (29)	29,081 (30)	13,687 (27)	23,695 (27)
Up to 4 years of college (MoBa) / Technical school (DNBC)	33,680 (41)	39,417 (40)	6,886 (14)	11,863 (14)
More than 4 years of college(MoBa)/High school or more(DNBC)	18,576 (23)	21,755 (22)	25,969 (52)	44,771 (52)
Missing	343		507	
Maternal smoking during pregnancy (n(%))				
No	62,939 (77)	75,005 (76)	37,954 (75)	63,743 (73)
During the first 12 weeks only	10,766 (13)	13,220 (14)	4,115 (8)	8,465 (10)
Also after the first 12 weeks	7,605 (9)	10,062 (10)	8,631(17)	14,582 (17)
Missing	320		1	
Maternal pre-pregnancy BMI (n(%))				
Underweight (<18.5)	2,363 (3)	3,115 (3)	2,066 (4)	3,911 (5)
Normal weight (18.5-24.9)	52,433 (66)	64,220 (65)	33,614 (67)	58,441 (67)
Overweight (25.0-29.9)	17,474 (22)	21,547 (22)	9,996 (20)	17,279 (20)
Obese (≥30.0)	7,375 (9)	9,405 (10)	4,211 (8)	7,159 (8)
Missing	1,985		814	
Maternal diabetes (n(%)) [b]				
No	81,268 (99.6)	97,829 (99.5)	49,316 (97)	84,460 (97)
Yes	362 (0.4)	458 (0.5)	1,385 (3)	2,329 (3)
Missing	0		0	
Paternal age (n(%))				
Less than 25	3,566 (4)	4,837 (5)	1,656 (3)	3,713 (4)
25-29	18,446 (23)	22,341 (23)	13,184 (26)	23,175 (27)
30-34	31,934 (39)	37,929 (39)	20,011 (40)	34,298 (40)
35+	27,492 (34)	33,180 (34)	15,232 (30)	25,602 (30)
Missing	192		618	

Paternal education (n(%)) [a]				
Less than high school (MoBa) / 9th grade with exam (DNBC)	8,026 (10)	10,819 (11)	9,430 (19)	16,881 (20)
High school (MoBa) / 10th grade with exam (DNBC)	31,845 (40)	39,981 (41)	17,608 (36)	31,068 (36)
Up to 4 years of college (MoBa) / Technical school (DNBC)	21,728 (27)	26,083 (26)	2,784 (6)	4,988 (6)
More than 4 years of college (MoBa)/ High school or more (DNBC)	17,609 (22)	21,404 (22)	18,928 (37)	33,851 (39)
Missing	2,422		1,951	
Paternal BMI (n(%))				
Underweight (<18.5)	163 (0.2)	223 (0.2)	191 (0.4)	674 (0.8)
Normal weight (18.5-24.9)	34,971 (45)	43,921 (45)	25,499 (53)	44,036 (51)
Overweight (25.0-29.9)	35,313 (45)	44,418 (45)	19,274 (40)	36,014 (42)
Obese (≥30.0)	7,544 (10)	9,724 (10)	3,475 (7)	6,065 (7)
Missing	3,639		2,262	
Paternal smoking during pregnancy (n(%))				
No	59,930 (74)	72,189 (73)	35,827 (71)	60,527 (70)
Yes	21,301 (26)	26,098 (27)	14,834 (29)	26,262 (30)
Missing	399		40	
Paternal type 1 diabetes (n(%))				
No	81,106 (99)	97,660 (99)	NA	NA
Yes	524 (0.6)	627 (0.6)	NA	NA
Child sex (n(%))				
Male	41,796 (51)	50,391 (51)	25,773 (51)	44,487 (51)
Female	39,834 (49)	47,896 (49)	24,928 (49)	42,302 (49)
Child birthweight in grams (Median(IQR))	3,620 (660)	3,603 (670)		3,600 (700)
Missing	99		251	
Child delivery by caesarean section (n(%))				
No	70,561 (86)	84,531 (86)	43,080 (85)	73,457 (85)
Yes	11,069 (14)	13,756 (14)	7,621 (15)	13,331 (15)
Uncertain	0		0	1 (0.0)
Child weight gain the first year of life in kg (Median(IQR))	6.3 (1.4)	6.3 (1.4)	6.6 (1.1)	6.6 (1.5)
Missing (n(%))	21,430 (26.3)		9,403 (18.5)	
Children with type 1 diabetes (n(%))				
No	81,337 (99.6)	97,938 (99.6)	50,495 (99.6)	86,449 (99.6)
Yes	293 (0.4)	349 (0.4)	206 (0.4)	340 (0.4)

BMI, body mass index; MoBa, The Norwegian Mother and Child Cohort Study; DNBC, The Danish National Birth Cohort; SD, Standard deviation.

[a] Note that the categories for parental education in the Norwegian Mother and Child Cohort study (MoBa) and the Danish National Birth Cohort (DNBC) are not directly comparable.

[b] Maternal diabetes includes only type 1 diabetes in MoBa, while it includes all forms of diabetes in DNBC.

eTable 2 Associations of parental smoking during pregnancy and childhood-onset type 1 diabetes in the Norwegian Mother and Child Cohort Study (MoBa) and the Danish National Birth Cohort (DNBC)

Study	Maternal smoking during pregnancy	Person years	Events	Confounder adjusted Model 1 [a] HR (95% CI)	Confounder and mediator adjusted Model 2 [b] HR (95% CI)	Confounder and mediator adjusted Model 3 [c] HR (95% CI)	Heterogeneity P-value
MoBa	No	812,149	276	1	1	1	
	First 12 gestational weeks	146,014	43	0.83 (0.59, 1.16)	0.83 (0.59, 1.16)	0.81 (0.58, 1.14)	
	After 12 gestational weeks	117,300	30	0.67 (0.45, 0.99)	0.67 (0.45, 0.99)	0.63 (0.43, 0.94)	
DNBC	No	904,916	263	1	1	1	
	First 12 gestational weeks	133,159	35	0.95 (0.66, 1.37)	0.95 (0.66, 1.37)	0.94 (0.66, 1.36)	
	After 12 gestational weeks	171,744	42	0.65 (0.46, 0.90)	0.65 (0.46, 0.91)	0.63 (0.45, 0.89)	
Combined	No	1,717,065	539	1	1	1	
	First 12 gestational weeks	279,173	78	0.88 (0.69, 1.13)	0.88 (0.69, 1.13)	0.87 (0.68, 1.11)	0.56
	After 12 gestational weeks	289,044	72	0.66 (0.51, 0.85)	0.66 (0.51, 0.85)	0.63 (0.49, 0.82)	1.00
Study	Paternal smoking during pregnancy	Person years	Events	Confounder adjusted Model 1 [a] HR (95% CI)	Confounder and mediator adjusted Model 2 [b] HR (95% CI)	Confounder and mediator adjusted Model 3 [c] HR (95% CI)	Heterogeneity P-value
MoBa	No	780,557	251	1	1	1	
	Yes	294,906	98	1.02 (0.79, 1.30)	1.01 (0.79, 1.30)	0.99 (0.78, 1.28)	
DNBC	No	862,634	242	1	1	1	
	Yes	347,185	98	0.93 (0.73, 1.18)	0.93 (0.73, 1.18)	0.93 (0.73, 1.18)	
Combined	No	1,643,191	493	1	1	1	
	Yes	642,091	196	0.97 (0.82, 1.16)	0.97 (0.81, 1.15)	0.96 (0.81, 1.14)	0.72

CI, confidence interval; HR, hazard ratio.

[a] Model 1: Maternal smoking during pregnancy adjusted for maternal age, parity, education, pre-pregnancy BMI and diabetes (maternal type 1 diabetes in MoBa and all types of maternal diabetes in DNBC); paternal smoking adjusted for paternal age, education, BMI and diabetes (MoBa only).
[b] Model 2: Adjusted for all the same characteristics as model 1 in addition to offspring sex, birthweight and delivery by caesarean section.
[c] Model 3: Adjusted for all the same characteristics as model 2 in addition to offspring weight gain the first year of life.

We imputed 20 datasets of missing exposure and covariate information by multiple imputation by chained equations, and run analyses with these. This analysis takes into account uncertainty in the imputation.

eTable 3 Analysis of parental smoking during pregnancy and childhood-onset type 1 diabetes in the Norwegian Mother and Child Cohort Study (MoBa) and the Danish National Birth Cohort (DNBC), restricted to those with complete data on covariates

Study	Maternal smoking during pregnancy	Person years	Events	Confounder adjusted Model 1 [a] HR (95% CI)	Confounder and mediator adjusted Model 2 [b] HR (95% CI)	Confounder and mediator adjusted Model 3 [c] HR (95% CI)	Heterogeneity P-value
MoBa	No	684,182	236	1	1	1	
	First 12 gestational weeks	119,727	35	0.73 (0.49, 1.07)	0.72 (0.49, 1.06)	0.69 (0.43, 1.10)	
	After 12 gestational weeks	89,203	21	0.53 (0.33, 0.86)	0.53 (0.33 to 0.86)	0.57 (0.32, 0.99)	
DNBC	No	563,505	167	1	1	1	
	First 12 gestational weeks	56,581	15	0.84 (0.49, 1.44)	0.86 (0.50, 1.47)	0.80 (0.43, 1.48)	
	After 12 gestational weeks	102,108	24	0.62 (0.40 to 0.96)	0.63 (0.40, 0.98)	0.64 (0.39, 1.03)	
Combined	No	1,247,687	403	1	1	1	
	First 12 gestational weeks	176,308	50	0.77 (0.56, 1.05)	0.77 (0.56, 1.05)	0.73 (0.50, 1.06)	0.71
	After 12 gestational weeks	191,311	45	0.58 (0.42, 0.80)	0.58 (0.42, 0.81)	0.61 (0.42, 0.88)	0.76
Study	Paternal smoking during pregnancy	Person years	Events	Confounder adjusted Model 1 [a] HR (95% CI)	Confounder and mediator adjusted Model 2 [b] HR (95% CI)	Confounder and mediator adjusted Model 3 [c] HR (95% CI)	
MoBa	No	650,935	211	1	1	1	
	Yes	241,533	81	0.98 (0.74, 1.31)	0.98 (0.73, 1.31)	1.04 (0.74, 1.45)	
DNBC	No	526,617	151	1	1	1	
	Yes	195,577	55	0.93 (0.68, 1.29)	0.95 (0.69, 1.31)	1.00 (0.71, 1.43)	
Combined	No	1,177,552	362	1	1	1	
	Yes	437,110	136	0.96 (0.77, 1.19)	0.97 (0.78, 1.20)	1.02 (0.80, 1.30)	0.87

CI, confidence interval; HR, hazard ratio.

[a] Model 1: Adjusted for maternal age, parity, education, pre-pregnancy BMI and diabetes (maternal type 1 diabetes in MoBa and all types of maternal diabetes in DNBC); paternal smoking was adjusted for paternal age, education, BMI and diabetes (MoBa only).

[b] Model 2: Adjusted for all the same characteristics as model 1 in addition to offspring sex, birthweight and delivery by caesarean section.

[c] Model 3: Adjusted for all the same characteristics as model 2 in addition to offspring weight gain the first year of life.

eTable 4 Analysis of the average number of cigarettes the mother smoked per day during pregnancy and childhood-onset type 1 diabetes in the Norwegian Mother and Child Cohort Study (MoBa) and the Danish National Birth Cohort (DNBC), restricted to those with complete data on covariates

Study	Average number of cigarettes smoked per day during pregnancy	Person years	Events	Adjusted Model 1 [a] HR (95% CI)	Adjusted Model 2 [b] HR (95% CI)	Adjusted Model 3 [c] HR (95% CI)	Heterogeneity P-value
MoBa	No	684,182	236	1	1	1	
	Unknown amount among smokers	125,101	36	0.72 (0.49, 1.05)	0.71 (0.48, 1.04)	0.69 (0.43, 1.09)	
	≤5 cigs	56,570	15	0.59 (0.33, 1.03)	0.58 (0.33, 1.03)	0 71 (0.38, 1.31)	
	6-10 cigs	20,950	4	0.45 (0.17, 1.22)	0.46 (0.17, 1.24)	0.16 (0.02, 1.16)	
	More than 10 cigs	6,308	1	0.37 (0.05, 2.64)	0.38 (0.05, 2.69)	0.52 (0.07, 3.66)	
DNBC	No	563,505	167	1	1	1	
	Unknown amount among smokers	82,948	21	0.59 (0.37, 0.94)	0.60 (0.38, 0.96)	0.53 (0.31, 0.93)	
	≤5 cigs	15,765	4	0.79 (0.29, 2.13)	0.80 (0.30, 2.17)	0.95 (0.35, 2.59)	
	6-10 cigs	27,465	6	0.62 (0.27, 1.40)	0.63 (0.27, 1.44)	0.64 (0.26, 1.58)	
	More than 10 cigs	32,511	8	1.34 (0.66, 2.73)	1.37 (0.67, 2.82)	1.48 (0.69, 3.15)	
Combined	No	1,247,687	403	1	1	1	
	Unknown amount among smokers	208,049	57	0.67 (0.50, 0.89)	0.66 (0.49, 0.89)	0.62 (0.43, 0.88)	0.47
	≤5 cigs	72,335	19	0.63 (0.39, 1.04)	0.63 (0.38, 1.03)	0.77 (0.46, 1.30)	0.63
	6-10 cigs	48,415	10	0.54 (0.29, 1.02)	0.55 (0.29, 1.05)	0.44 (0.13, 1.47)	0.22
	More than 10 cigs	38,819	9	1.00 (0.34, 2.88)	1.02 (0.36, 2.93)	1.29 (0.64, 2.63)	0.33

CI, confidence interval; HR, hazard ratio.

[a] Model 1: Adjusted for maternal age, parity, education, pre-pregnancy BMI and diabetes (maternal type 1 diabetes in MoBa and all types of maternal diabetes in DNBC); paternal smoking was adjusted for paternal age, education, BMI and diabetes (MoBa only).

[b] Model 2: Adjusted for all the same characteristics as model 1 in addition to offspring sex, birthweight and delivery by caesarean section.

[c] Model 3: Adjusted for all the same characteristics as model 2 in addition to offspring weight gain the first year of life.

eTable 5 Analysis of environmental tobacco smoke exposure during the first 6 months of life and childhood-onset type 1 diabetes in the Norwegian Mother and Child Cohort Study (MoBa) and the Danish National Birth Cohort (DNBC), restricted to those with complete data on covariates

Study	Maternal smoking at child age 0-6 months	Person years	Events	Adjusted [a] HR (95% CI)	Heterogeneity P-value
MoBa	No	704,573	249	1	
	Yes	117,600	28	0.78 (0.46, 1.34)	
DNBC	No	613,087	179	1	
	Yes	109,107	27	0.87 (0.49, 1.55)	
Combined	No	1,317,660	428	1	
	Yes	226,707	55	0.82 (0.56, 1.21)	0.79
Study	Household smoking at child age 0-6 months	Person years	Events	Adjusted [b] HR (95% CI)	Heterogeneity P-value
MoBa	No	820,339	274	1	
	Yes	30,033	13	1.34 (0.76, 2.36)	
DNBC	No	639,692	184	1	
	Yes	82,502	22	1.07 (0.63, 1.83)	
Combined	No	1,460,031	458	1	
	Yes	112,535	35	1.19 (0.81, 1.75)	0.57

CI, confidence interval; HR, hazard ratio.

[a] Adjusted for maternal smoking during pregnancy, age, parity, education, diabetes (maternal type 1 diabetes in MoBa and all types of maternal diabetes in DNBC), and pre-pregnancy BMI.

[b] Adjusted for parental (both maternal and paternal) age, education, diabetes (maternal type 1 diabetes in MoBa and all types of maternal diabetes in DNBC), BMI and smoking during pregnancy, in addition to maternal parity.

eTable 6 Associations of paternal smoking the first 6 months of the child’s life and childhood-onset type 1 diabetes in the Norwegian Mother and Child Cohort Study

Complete-case analysis			
Paternal smoking at child age 0-6 months	Person years	Events	Adjusted [a] HR (95% CI)
No	639,237	214	1
Yes	18,6960	63	0.81 (0.48, 1.35)
Multiple imputation analysis			
Paternal smoking at child age 0-6 months	Person years	Events	Adjusted [a] HR (95% CI)
No	791,833	269	1
Yes	235,194	80	0.91 (0.55, 1.51)

CI, confidence interval; HR, hazard ratio.

[a] Adjusted for paternal age, education, diabetes, BMI and smoking during pregnancy.

eTable 7 The distribution of characteristics in the Norwegian register-based cohort (n= 434,627)

Characteristics	Mean(SD) / N(%)
Maternal age	29.7 (5.2)
Maternal parity	
0	181,684 (41.8)
1	155,822 (35.9)
2	68,996 (15.9)
3 or higher	28,125 (6.5)
Maternal education	
Primary	70,914 (16.3)
Secondary	127,696 (29.4)
Tertiary	220,334 (50.7)
Missing	15,683 (3.6)
Maternal type 1 diabetes	
No	429,354 (98.8)
Yes	5,273 (1.2)
Maternal smoking	
No	373,718 (86.0)
Quit during pregnancy	24,426 (5.6)
Missing	36,483 (8.4)
Child sex	
Male	223,211 (51.4)
Female	211,416 (48.6)
Child birthweight (grams)	3,508 (592)

eTable 8 Association between maternal smoking during pregnancy and childhood-onset type 1 diabetes in a Norwegian register-based cohort (n=434,627)

Maternal smoking	Person years	Events	Confounder adjusted Model 1 [a]<br>HR (95% CI)	Confounder and mediator adjusted Model 2 [b]<br>HR (95% CI)
No	2,347,735	605	1	1
Quit during pregnancy	172,222	43	0.88 (0.64, 1.21)	0.88 (0.64, 1.21)
Still smoking at the end of pregnancy	245,999	44	0.65 (0.47, 0.89)	0.66 (0.48, 0.91)

CI, confidence interval; OR, odds ratio.

[a] Model 1: Adjusted for maternal age, parity, education, and insulin-treated diabetes.

[b] Model 2: Adjusted for all the same characteristics as model 1, in addition to offspring sex, birthweight and delivery by caesarean section.

eTable 9 Associations of cord blood cotinine levels and risk of type 1 diabetes in the case-control study nested in the Norwegian Mother and Child Cohort Study

Cotinine concentration	N cases	N controls	Confounder adjusted Model 1 [a] OR (95% CI)	Confounder and Mediator adjusted Model 2 [b] OR (95% CI)	Confounder and mediator adjusted Model 3 [c] OR (95% CI)
Not detected	131	391	1	1	1
<30 nmol/L	15	44	0.82 (0.42 to 1.58)	0.81 (0.42 to 1.59)	0.82 (0.39 to 1.76)
≥30 nmol/L	8	41	0.42 (0.17 to 1.01)	0.43 (0.18 to 1.02)	0.58 (0.21 to 1.57)

CI, confidence interval; OR, odds ratio.

[a] Model 1: Adjusted for maternal age, parity, education, pre-pregnancy body-mass index and type 1 diabetes.

[b] Model 2: Adjusted for all the same characteristics as model 1 in addition to offspring sex and birthweight, and caesarian section.

[c] Model 3: Adjusted for the same characteristics as model 2 in addition to HLA genotype. HLA genotypes were categorized into two groups based on established risk for type 1 diabetes: increased risk (at least one copy of HLA DQA1*03-DQB1*03:02 [DQ8-DR4] or DQA1*05:01-DQB1*02:01 [DQ2-DR3], and no protective HLA-DQA1*01:02-DQB1*06:02 haplotype) or not increased risk (any other genotype).

We imputed 20 datasets of missing exposure and covariate information by multiple imputation by chained equations, and run analyses with these. This analysis takes into account uncertainty in the imputation.

eTable 10 Associations of cord blood cotinine and risk of type 1 diabetes in the case-control study nested in the Norwegian Mother and Child Cohort Study, restricted to those with complete data on covariates

Cotinin concentration	N cases	N controls	Confounder adjusted Model 1 [a] OR (95% CI)	Confounder and Mediator adjusted Model 2 [b] OR (95% CI)	Confounder and mediator adjusted Model 3 [c] OR (95% CI)
Not detected	131	391	1	1	1
<30 nmol/L	15	44	0.82 (0.42, 1.60)	0.81 (0.41, 1.60)	0.95 (0.44, 2.07)
≥30 nmol/L	8	41	0.38 (0.15, 0.97)	0.39 (0.15, 0.99)	0.53 (0.18, 1.56)

CI, confidence interval; OR, odds ratio.
[a] Model 1: Adjusted for maternal age, parity, education, pre-pregnancy body-mass index and type 1 diabetes.
[b] Model 2: Adjusted for all the same characteristics as model 1 in addition to offspring sex and birthweight, and caesarian section.
[c] Model 3: Adjusted for the same characteristics as model 2 in addition to HLA genotype. HLA genotypes were categorized into two groups based on established risk for type 1 diabetes: increased risk (at least one copy of HLA DQA1*03-DQB1*03:02 [DQ8-DR4] or DQA1*05:01-DQB1*02:01 [DQ2-DR3], and no protective HLA-DQA1*01:02-DQB1*06:02 haplotype) or not increased risk (any other genotype).

eFigure 1 Illustration of sample selection the two Scandinavian pregnancy cohorts

(a) The Norwegian Mother and Child Cohort Study (b) the Danish National Birth Cohort (DNBC)

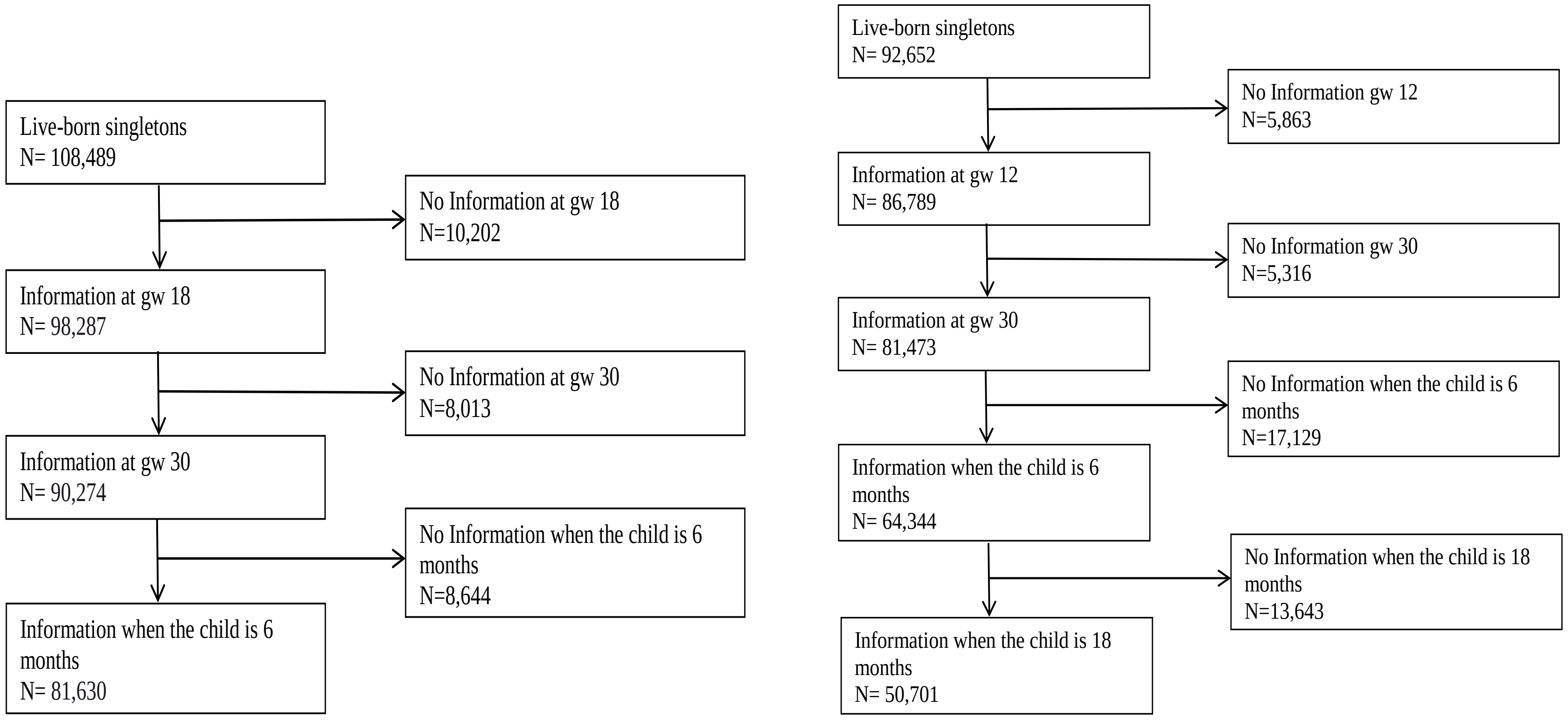

We restricted our analysis to children of mothers who had provided information on maternal smoking throughout pregnancy in both MoBa (questionnaires administered at gw 18, gw 30 and 6 months after delivery) and DNBC (questionnaire administered at gw 12, gw 30 and 6 months after delivery). For both cohorts, the questionnaire administered 6 months after delivery included retrospective information on maternal smoking during the last part of pregnancy, in addition to exposure to maternal and household smoking during the first 6 months of life. For DNBC, we restricted the analysis to those who had information from the questionnaire administered 18 months after delivery, since this questionnaire provided important information on paternal background characteristics.

eFigure 2 Kaplan-Meier plots showing the risk of type 1 diabetes by maternal smoking during pregnancy in the two pregnancy cohorts

(a) The Norwegian Mother and Child Cohort Study (MoBa) (b) the Danish National Birth Cohort (DNBC)

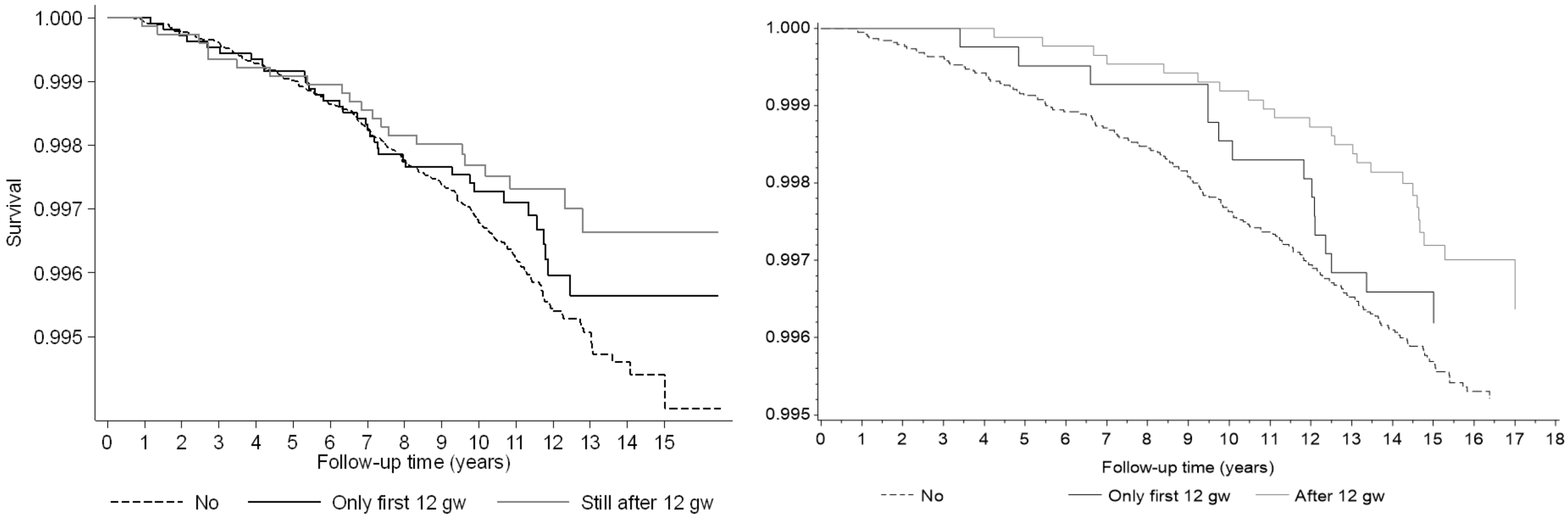

eFigure 3 Kaplan-Meier plot showing the risk of type 1 diabetes by maternal smoking during pregnancy in the Norwegian register-based cohort

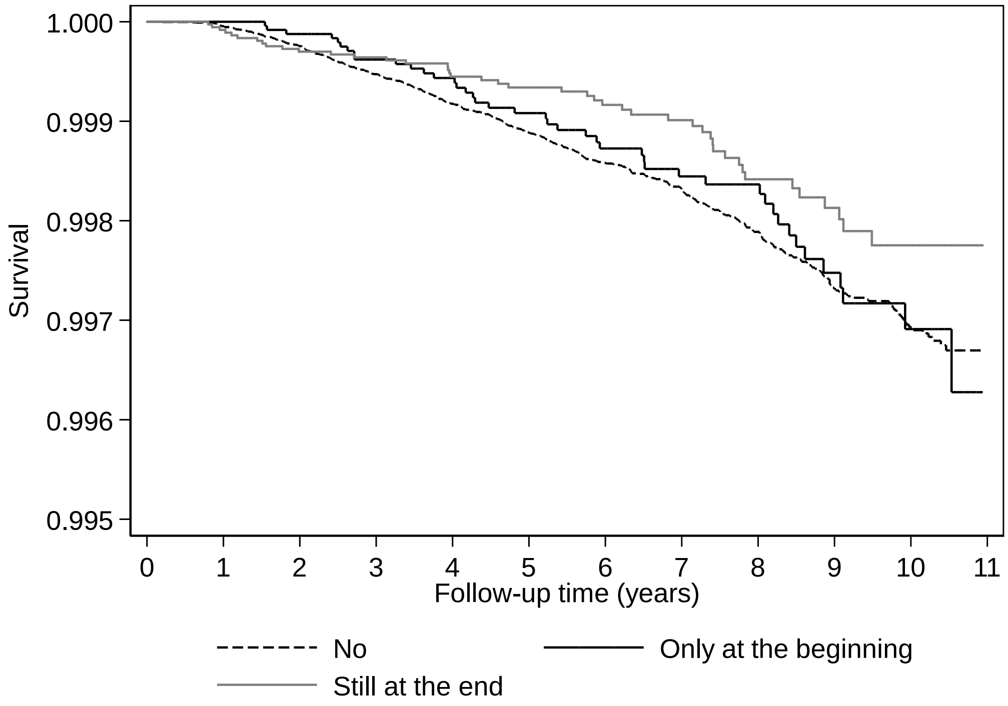

eFigure 4 Illustration of sample selection in the case-control study of cotinine nested in the Norwegian Mother and Child Cohort Study

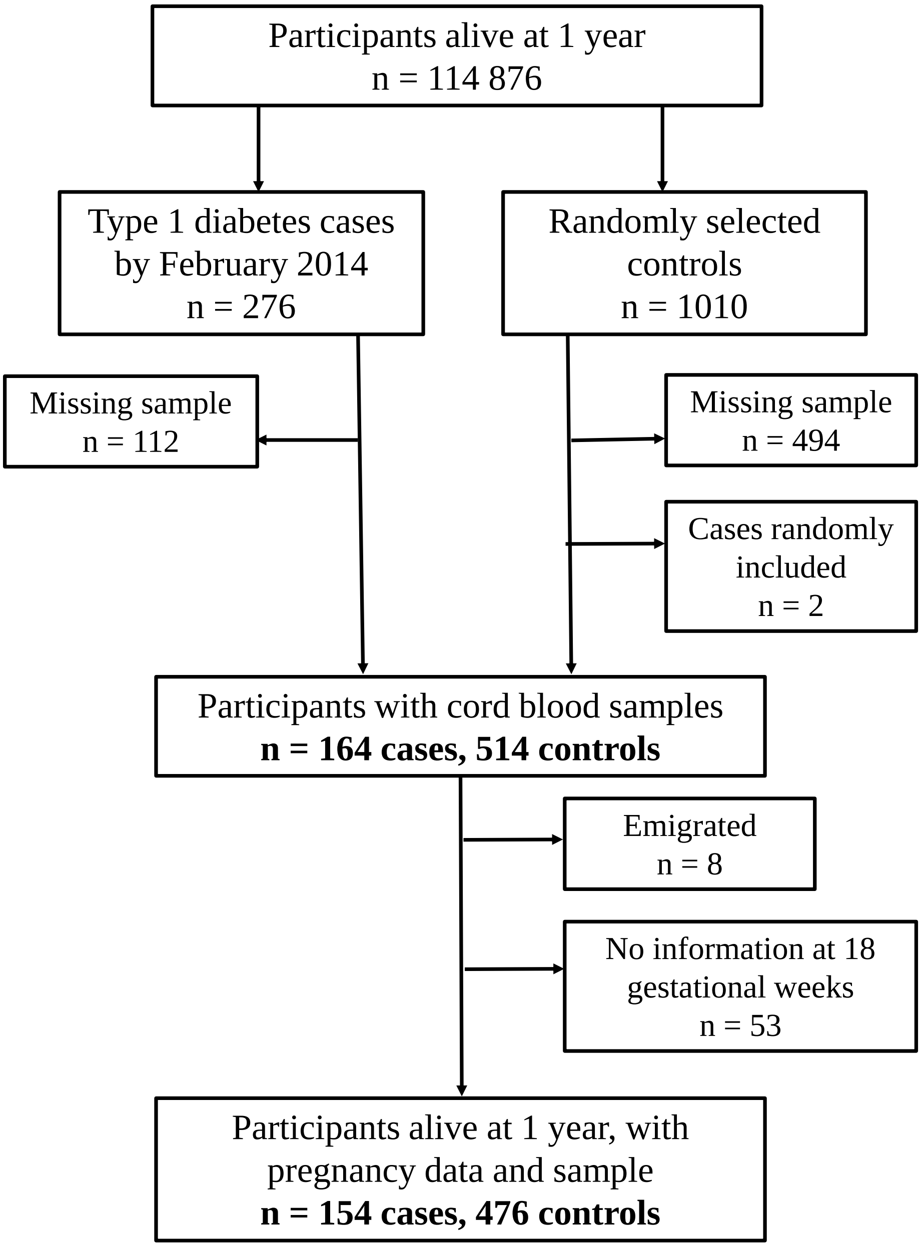

Supplement: Supplementary file 1 [file ede-29-848-s001.pdf]
